# Interaction of Mycotoxins with α_1_-Acid Glycoprotein (AGP) and Bovine Milk Proteins: Zearalenone, Zearalenols, and Sterigmatocystin Form Highly Stable Complexes with AGP

**DOI:** 10.3390/toxins17040151

**Published:** 2025-03-21

**Authors:** Miklós Poór, Patrik Gömbös, András Szabó, Balázs Zoltán Zsidó, Csaba Hetényi, Tamás Huber, András Lukács, Sándor Kunsági-Máté

**Affiliations:** 1 Department of Laboratory Medicine, Medical School, University of Pécs, Ifjúság útja 13, H-7624 Pécs, Hungary; 2 Molecular Medicine Research Group, János Szentágothai Research Centre, University of Pécs, Ifjúság útja 20, H-7624 Pécs, Hungary; 3 Institute of Physiology and Nutrition, Department of Physiology and Animal Health, Agribiotechnology and Precision Breeding for Food Security National Laboratory, Hungarian University of Agriculture and Life Sciences, H-2103 Gödöllő, Hungary; gombos.patrik@uni-mate.hu (P.G.); szabo.andras@uni-mate.hu (A.S.); 4 HUN-REN-MATE Mycotoxins in the Food Chain Research Group, Hungarian University of Agriculture and Life Sciences, Guba Sándor u. 40, H-7400 Kaposvár, Hungary; 5 Pharmacoinformatics Unit, Department of Pharmacology and Pharmacotherapy, Medical School, University of Pécs, Szigeti út 12, H-7624 Pécs, Hungary; zsido.balazs@pte.hu (B.Z.Z.); hetenyi.csaba@pte.hu (C.H.); 6 National Laboratory for Drug Research and Development, H-1117 Budapest, Hungary; 7 Department of Biophysics, Medical School, University of Pécs, Szigeti út 12, H-7624 Pécs, Hungary; tamas.huber@aok.pte.hu (T.H.); andras.lukacs@aok.pte.hu (A.L.); 8 Department of Organic and Medicinal Chemistry, Faculty of Pharmacy, University of Pécs, Honvéd u. 1, H-7624 Pécs, Hungary; kunsagi-mate.sandor@gytk.pte.hu; 9 Green Chemistry Research Group, János Szentágothai Research Centre, University of Pécs, Ifjúság útja 20, H-7624 Pécs, Hungary

**Keywords:** mycotoxins, zearalenone, zearalenols, sterigmatocystin, aflatoxins, α_1_-acid glycoprotein, casein, β-lactoglobulin, α-lactalbumin, bovine serum albumin

## Abstract

Mycotoxins are frequent food contaminants posing health risk to humans and animals. Since these interactions have been barely studied yet, we examined the potential complex formation of mycotoxins with human α_1_-acid glycoprotein (AGP) and with bovine milk proteins (including casein (CSN), β-lactoglobulin (LG), and α-lactalbumin (LA)) based on fluorescence spectroscopic and ultracentrifugation techniques. Only weak interactions (log*K* = 2.7 to 3.5) of certain mycotoxins were observed with CSN, LG, and/or LA. Ultracentrifugation experiments demonstrated that aflatoxin M1, zearalenone, and α-zearalenol form more stable complexes with CSN than with LG or LA. These mycotoxins bound to bovine serum albumin with more than a tenfold higher affinity compared to CSN; nevertheless, it has likely limited importance due to the relatively low levels of BSA in bovine milk. Zearalenone, zearalenols, and sterigmatocystin showed strong interactions with AGP (log*K* = 5.5 to 6.4), suggesting that AGP may play an important role in the plasma protein binding of these mycotoxins.

## 1. Introduction

Mycotoxins, the toxic secondary metabolites of molds, are common food contaminants [1]. Most frequently, *Aspergillus* and/or *Penicillium* strains are responsible for the production of aflatoxin B1 (AFB1), sterigmatocystin (STC), cyclopiazonic acid (CPA), citrinin (CIT), ochratoxin A (OTA), and patulin (PAT) [1]. Typically, *Fusarium* molds are the sources of the mycotoxins deoxynivalenol (DON), fumonisin B1 (FB1), T-2 toxin (T2), and zearalenone (ZEN) [2]. The high and/or chronic exposure to mycotoxins poses a serious health risk, leading to the possible development of nephrotoxic, hepatotoxic, immunotoxic, neurotoxic, endocrine disruptor, teratogenic, and/or carcinogenic effects [1]. In addition to cereals, bakery products, fruits, vegetables, oilseeds, and spices [1], mycotoxins can also contaminate milk and dairy products [3,4,5] via carry-over, entering the human food chain [6]. For example, in bovine milk, the frequent appearance of aflatoxin M1 (AFM1), OTA, ZEN, and/or α-zearalenol (α-ZEL) has been reported [3,4,5].

Some mycotoxins (e.g., OTA, ZEN, and alternariol) bind to serum albumins with high affinity, including human serum albumin (HSA) and bovine serum albumin (BSA) [7,8,9,10]. However, we did not find any data regarding the potential interaction of mycotoxins with α_1_-acid glycoprotein (AGP). AGP (also known as orosomucoid; molecular weight ≈ 43 kDa) is an acute-phase protein containing five asparaginyl-linked glycan chains [11]. AGP is one of the most abundant proteins in the circulation, its typical plasma concentration is approximately 0.6–1.2 g/L (≈14–28 μM) in humans [11]. AGP forms highly stable complexes with certain drugs (e.g., imatinib and vismodegib), strongly affecting their pharmacokinetics [12].

Caseins (CSN) and whey proteins (including β-lactoglobulin (LG), α-lactalbumin (LA), and BSA) are the most abundant protein components in bovine milk, which typically contains 30–36 g/L of total protein [13]. CSN (molecular weight ≈ 20–25 kDa) makes up approximately 80% of milk proteins [14]. LG (molecular weight ≈ 18 kDa) represents 50% of whey proteins and 12% of the total protein of milk, while LA (molecular weight ≈ 14 kDa) provides approximately 20% of the whey proteins and 3.5% of total protein [14]. Furthermore, relatively low amounts of BSA (molecular weight ≈ 66 kDa) are also presented in bovine milk (approximately 8% of whey proteins and 1.5% of the total protein) [15]. An earlier report described the interaction of AFM1 with CSN based on equilibrium dialysis [16]. Furthermore, spectroscopic and/or modeling studies suggested the complex formation of AFM1 with LA [17] and LG [18]. Based on some reports, AFM1 is cumulated in the whey fraction during the cheese making process, which may result from the formation of more stable complexes of this mycotoxin with whey proteins (e.g., LG and/or LA) compared to CSN [19,20]. Nevertheless, the interaction of mycotoxins with CSN, LG, and LA has been barely examined yet.

In the present work, we aimed to investigate the potential interactions of mycotoxins (AFB1, STC, CPA, CIT, OTA, PAT, DON, FB1, T2, ZEN, and some of their metabolites) with AGP, CSN, LG, and LA applying fluorescence spectroscopic and ultracentrifugation studies. This topic seems to be interesting because the formation of highly stable mycotoxin–AGP complexes may influence the toxicokinetics of mycotoxins, while the interaction of mycotoxins with milk proteins may affect their secretion into milk and/or their higher appearance in cheese or in whey-based food products. In addition, the identification of highly stable mycotoxin–protein complexes may help to find novel mycotoxin binder affinity proteins.

## 2. Results and Discussion

### 2.1. Interaction of Mycotoxins with α_1_-Acid Glycoprotein

**Figure 1:**
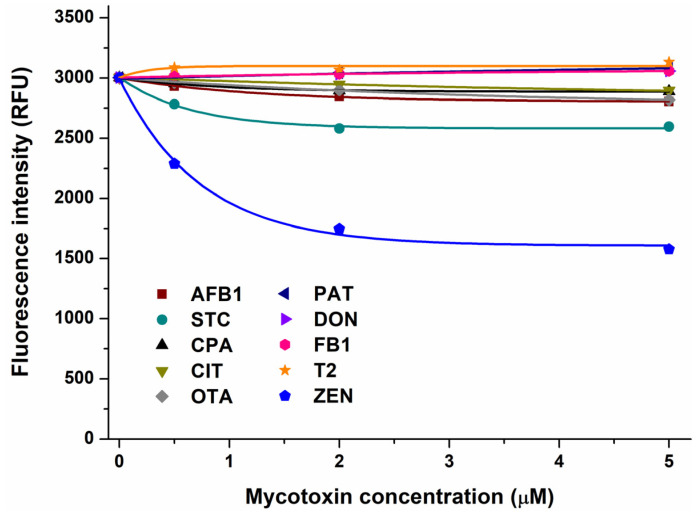
Effect of mycotoxins (0, 0.5, 2, and 5 μM) on fluorescence emission signal of AGP (1 μM) in PBS (pH 7.4; λ_ex_ = 285 nm, λ_em_ = 337 nm). Inner-filter effects of mycotoxins have been corrected (AFB1, aflatoxin B1; STC, sterigmatocystin; CPA, cyclopiazonic acid; CIT, citrinin; OTA, ochratoxin A; PAT, patulin; DON, deoxynivalenol; FB1, fumonisin B1; T2, T-2 toxin; and ZEN, zearalenone).

In the first experiment, the impacts of mycotoxins were examined on the emission signal of AGP (see the representative emission spectra in Figure S1). After the correction of the inner-filter effects of mycotoxins, we noticed no or only minor decreases in the fluorescence of the protein in the presence of AFB1, CPA, CIT, OTA, PAT, DON, FB1, and T2 (Figure 1). Therefore, these mycotoxins likely do not form or form only low-affinity complexes with AGP. However, STC and ZEN caused larger, concentration-dependent decreases in the emission signal of AGP, showing impacts even at a 0.5 μM concentration (Figure 1). These data suggest that STC and ZEN may form stable complexes with AGP; therefore, these interactions have been further characterized.

**Figure 2:**
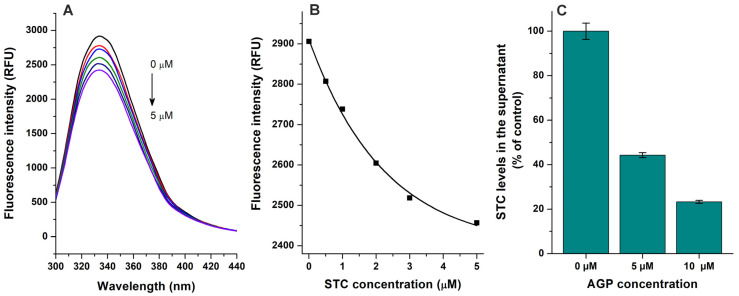
Interaction of sterigmatocystin (STC) with α_1_-acid glycoprotein (AGP) based on fluorescence quenching and ultracentrifugation experiments. (**A**) Representative emission spectra of AGP (1 μM) in presence of increasing STC concentrations (0, 0.5, 1, 2, 3, and 5 μM) in PBS (pH 7.4; λ_ex_ = 285 nm). (**B**) STC-induced decrease in emission signal of AGP (λ_em_ = 337 nm; inner-filter effect of STC has been corrected). (**C**) STC (2 μM) levels in supernatants after ultracentrifugation in absence and presence of AGP (5 and 10 μM) in PBS (pH 7.4).

Based on fluorescence quenching experiments (Figure 2A,B), the binding constant of the STC–AGP complex was calculated using the Hyperquad2006 software with non-linear fitting (Figure S2). Furthermore, the interaction of STC with AGP was also evaluated using ultracentrifugation studies, where AGP and the AGP-bound ligand molecules were sedimented then the free (unbound) concentration of STC was quantified from the supernatant [21,22]. AGP induced concentration-dependent decreases in the free fraction of STC (Figure 2C), confirming the formation of STC–AGP complexes. Log*K* values determined based on fluorescence quenching and ultrafiltration techniques are demonstrated in Table 1. These data suggest that the binding constant of STC–AGP is around 4 × 10^5^ L/mol, which highly exceeds the previously reported *K* value of the STC–HSA complex (≈2 × 10^4^ L/mol) [21]. Also, considering the significantly higher binding capacity of HSA (plasma level ≈ 500–750 μM) vs. AGP (plasma level ≈ 14–28 μM) in the human circulation, the STC–AGP interaction likely has toxicokinetic importance.

**Figure 3:**
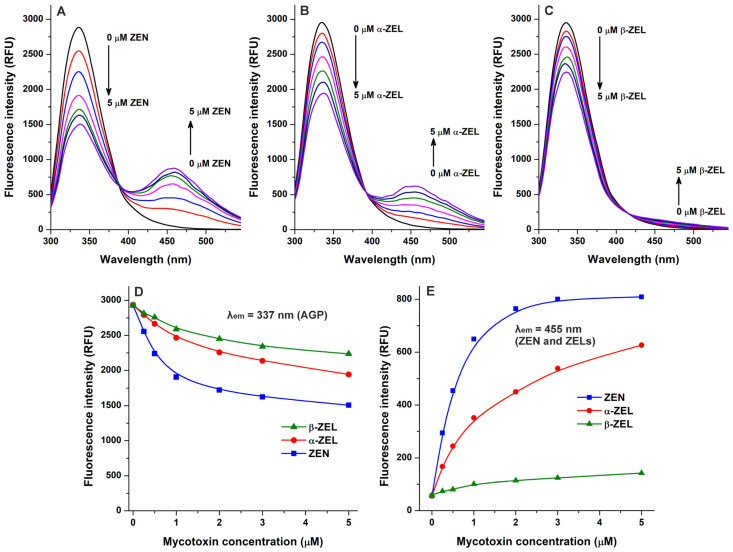
Interactions of zearalenone (ZEN), α-zearalenol (α-ZEL), and β-zearalenol (β-ZEL) with α_1_-acid glycoprotein (AGP) based on fluorescence spectroscopic studies. Representative emission spectra of AGP (1 μM) in the presence of increasing ZEN (**A**), α-ZEL (**B**), and β-ZEL (**C**) concentrations (each 0, 0.25, 0.5, 1, 2, 3, and 5 μM) in PBS (pH 7.4; λ_ex_ = 285 nm). (**D**) ZEN/ZEL-induced decreases in the emission signal of AGP (λ_em_ = 337 nm; the inner-filter effect of ZEN/ZELs has been corrected). (**E**) Emission signals of ZEN and ZELs (each 0–5 μM) at 455 nm in the presence of AGP (1 μM; λ_ex_ = 285 nm).

Because quenching studies suggested the interaction of ZEN with AGP (Figure 1), we also tested the complex formation of its reduced metabolites, α-ZEL and β-zearalenol (β-ZEL), with the protein. Similarly to ZEN, ZELs also reduced the emission signal of AGP at 337 nm in a concentration-dependent fashion (Figure 3A–D). Furthermore, gradually increasing second peaks appeared around 455 nm, which are produced by the intrinsic fluorescence of these mycotoxins [23,24]. Nevertheless, under the applied conditions, ZEN and ZELs alone (without AGP) showed negligible emission signals at 455 nm, while the increasing concentrations of these mycotoxins produced a saturation type concentration–intensity curve in the presence of AGP (Figure 3E). Considering these observations, the interaction of ZEN and ZELs with AGP can significantly enhance their fluorescence; which is in agreement with the previous observation that the complex formation of ZEN with HSA also strongly elevated the emission signal of the mycotoxin [23].

**Figure 4:**
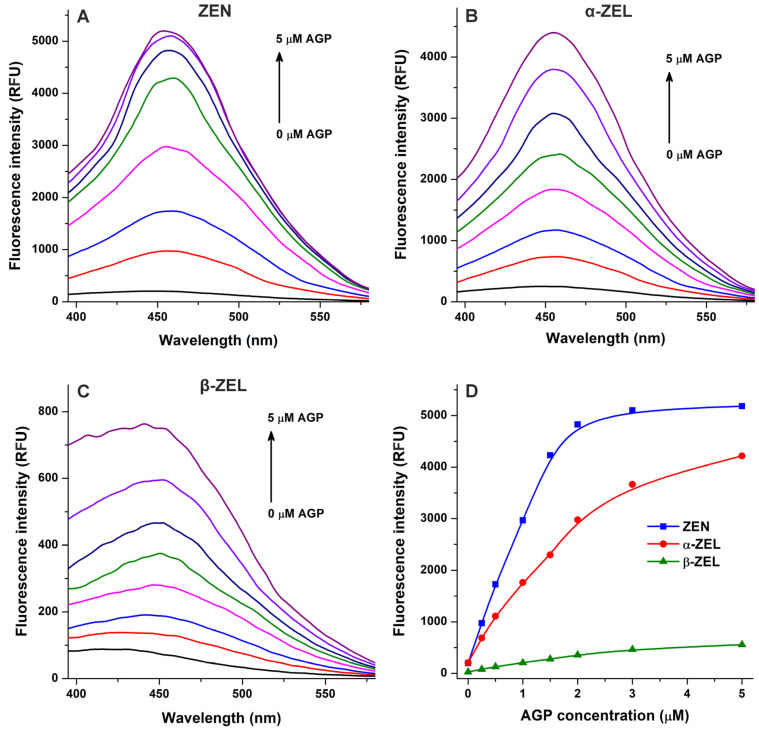
Effects of α_1_-acid glycoprotein (AGP) on the fluorescence emission signals of zearalenone (ZEN), α-zearalenol (α-ZEL), and β-zearalenol (β-ZEL). Representative emission spectra of ZEN (**A**), α-ZEL (**B**), and β-ZEL (**C**) (each 1 μM) in the presence of increasing AGP concentrations (0, 0.25, 0.5, 1, 1.5, 2, 3, and 5 μM) in PBS (pH 7.4; λ_ex_ = 315 nm). (**D**) AGP-induced increases in the emission signals of ZEN and ZELs (λ_em_ = 455 nm).

In the following experiment, we examined ZEN–AGP and ZEL–AGP interactions based on protein-induced fluorescence enhancement. The emission spectra of ZEN, α-ZEL, and β-ZEL (each 1 μM) were collected in the presence of increasing concentrations of AGP (0–5 μM; λ_ex_ = 315 nm). Under these circumstances, AGP exerted only minor background fluorescence at 455 nm, which were corrected before data evaluation. AGP strongly increased the emission signals of ZEN and ZELs at 455 nm (Figure 4), showing saturation type concentration–intensity curves and confirming again the formation of mycotoxin–AGP complexes. Furthermore, the emission signal of ZEN reached its plateau in the presence of 3 μM of AGP (Figure 3E and Figure 4D), suggesting the stronger interactions of ZEN with the protein compared to α-ZEL and β-ZEL.

**Figure 5:**
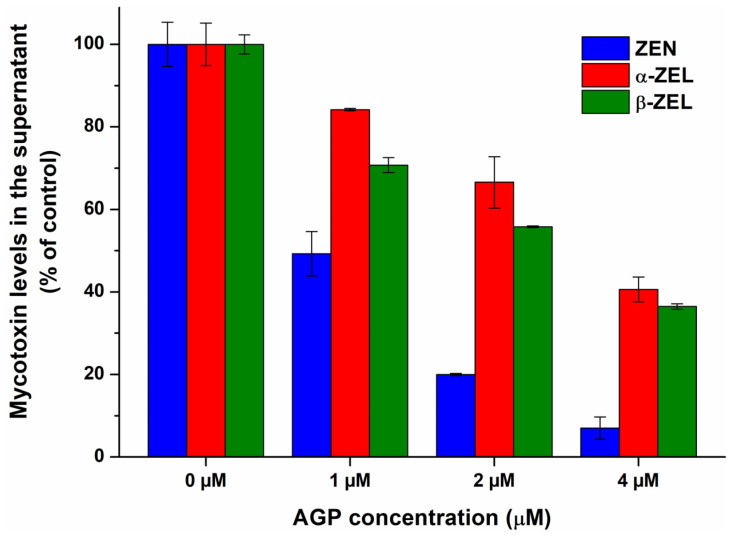
The interaction of α_1_-acid glycoprotein (AGP) with zearalenone (ZEN), α-zearalenol (α-ZEL), and β-zearalenol (β-ZEL) based on ultracentrifugation studies. ZEN and ZEL (each 1 μM) levels in the supernatant after ultracentrifugation in the absence and presence of AGP (1, 2, and 4 μM) in PBS (pH 7.4).

To confirm the results of spectroscopic studies, the complexation of ZEN and ZELs with AGP were also evaluated based on ultracentrifugation experiments. AGP considerably reduced the free fraction of ZEN and ZELs, inducing the strongest decrease in ZEN levels, followed by β-ZEL and α-ZEL (Figure 5). These data provide direct evidence regarding the formation of ZEN–AGP and ZEL–AGP complexes, and it proves our hypothesis that ZEN binds to AGP with higher affinity than its reduced metabolites.

Assuming a 1:1 stoichiometry of the complex formation, binding constants were determined based on fluorescence quenching and fluorescence enhancement with the Hyperquad2006 software (Figures S2 and S3), as well as based on ultracentrifugation experiments (Equation (2); Figure 5). These data suggest that the *K* values of α-ZEL–AGP and β-ZEL–AGP complexes are approximately 3 × 10^5^ L/mol and 6 × 10^5^ L/mol, respectively (Table 1). Both fluorescence spectroscopic and ultracentrifugation studies demonstrated the stronger interaction of ZEN with AGP compared to ZELs; nevertheless, the quenching study seems to underestimate the binding affinity of this mycotoxin (Table 1). Based on the AGP-induced enhancement in the emission signal of ZEN and the ultracentrifugation experiment, the binding constant of the ZEN–AGP complex is approximately 2 × 10^6^ L/mol. Importantly, ultracentrifugation studies provide the most reliable data, because we can directly quantify the free concentration of the mycotoxin from the protein-free supernatant, while fluorescence quenching is an indirect technique with several limitations, thus it provides an approximation with lower accuracy. Furthermore, in addition to the inner-filter effect and static quenching, other processes (e.g., Förster resonance energy transfer and collisional quenching) may also induce decreases in the emission signal of the protein, which have not been examined in the current study.

As it has been reported, ZEN forms stable complexes with HSA (log*K* = 5.1), and its reduced derivatives, α-ZEL (log*K* = 4.7) and β-ZEL (log*K* = 4.3), bind to this protein with lower affinity [9]. Even if we consider the much higher concentration of HSA vs. AGP in human circulation, ZEN, α-ZEL, and β-ZEL bind to AGP with approximately 15-fold, 5-fold, and 30-fold higher affinity compared to HSA, respectively. Therefore, AGP may affect the plasma protein binding and the toxicokinetics of ZEN and ZELs. It is also important to note that AGP is a positive acute phase protein; thus, its plasma levels (and binding capacity) can be considerably elevated as a result of inflammation, cancer, tissue injury, and/or trauma [12].

From another point of view, AGP can be considered as an affinity protein of ZEN. For example, BSA was successfully applied for the extraction of OTA from wine samples [25] and AOH from tomato juice [26], where the binding constants of OTA–BSA (log*K* = 6.5) and AOH–BSA (log*K* = 5.9) complexes [8,10] were similar to ZEN–AGP (Table 1).

**Figure 6:**
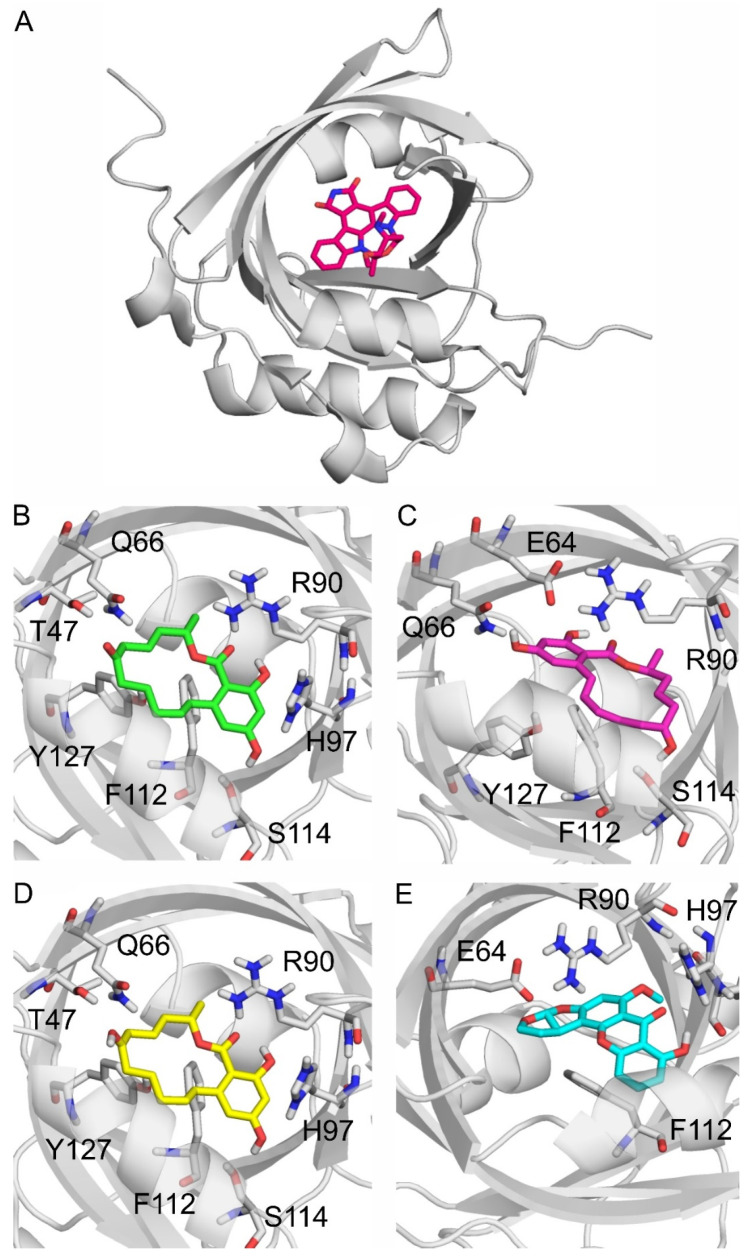
Alpha-1-acid glycoprotein (AGP; gray cartoon) and its binding site highlighted with an experimental co-crystalized ligand UCN-01 (red sticks) as seen in PDB:7oub (**A**). The binding modes of zearalenone ((**B**); ZEN), α-zearalenol ((**C**); α-ZEL), β-zearalenol ((**D**); β-ZEL), and sterigmatocystin ((**E**); and STC) to AGP, where the protein, the ligands, and the interacting amino acids are demonstrated as gray cartoons, colored sticks, and gray sticks, respectively. The interacting amino acids are labeled according to the numbering of PDB:7oub.

In modeling studies, UCN-01, the co-crystalized ligand of AGP (PDB:7oub, Figure 6A), was re-docked for validation purposes. The resulting RMSD of the re-docked binding mode was 0.559 Å in the first rank, which is an excellent match to the crystallographic binding mode (Figure S4). Then, the same docking protocol (see in Section 4.6) was applied to produce the binding modes of ZEN, α-ZEL, β-ZEL, and STC. The docked, first-ranked binding modes of ZEN and β-ZEL are practically identical (Figure 6B,D), whereas the binding mode of α-ZEL is different (Figure 6C). Compared to β-ZEL, a 90° turn around the longitudinal axis of the molecule is observed in the binding mode of α-ZEL, resulting in their slightly different interactions with the target. It may explain the lower affinity of α-ZEL toward AGP compared to ZEN or β-ZEL (Table 1). The two common amino acids in the binding of ZEN, α-ZEL, β-ZEL, and STC were R90 and F112. R90 interacts with the hydrophilic moieties of these mycotoxins, and F112 interacts with the ring system of the ligands through π-π interactions. Further hydrophilic interactions were observed with Q66 and H97 regarding ZEN and β-ZEL (Figure 6B,D), and with E64 for α-ZEL and STC (Figure 6C,E). ZEN had the most favorable ΔG_b_, followed by similar values for ZELs, then STC had the least favorable ΔG_b_ among the four mycotoxins. However, the ΔG_b_ difference between the strongest and weakest interactions was only 0.5 kcal/mol.

### 2.2. Interaction of Mycotoxins with Milk Proteins

**Figure 7:**
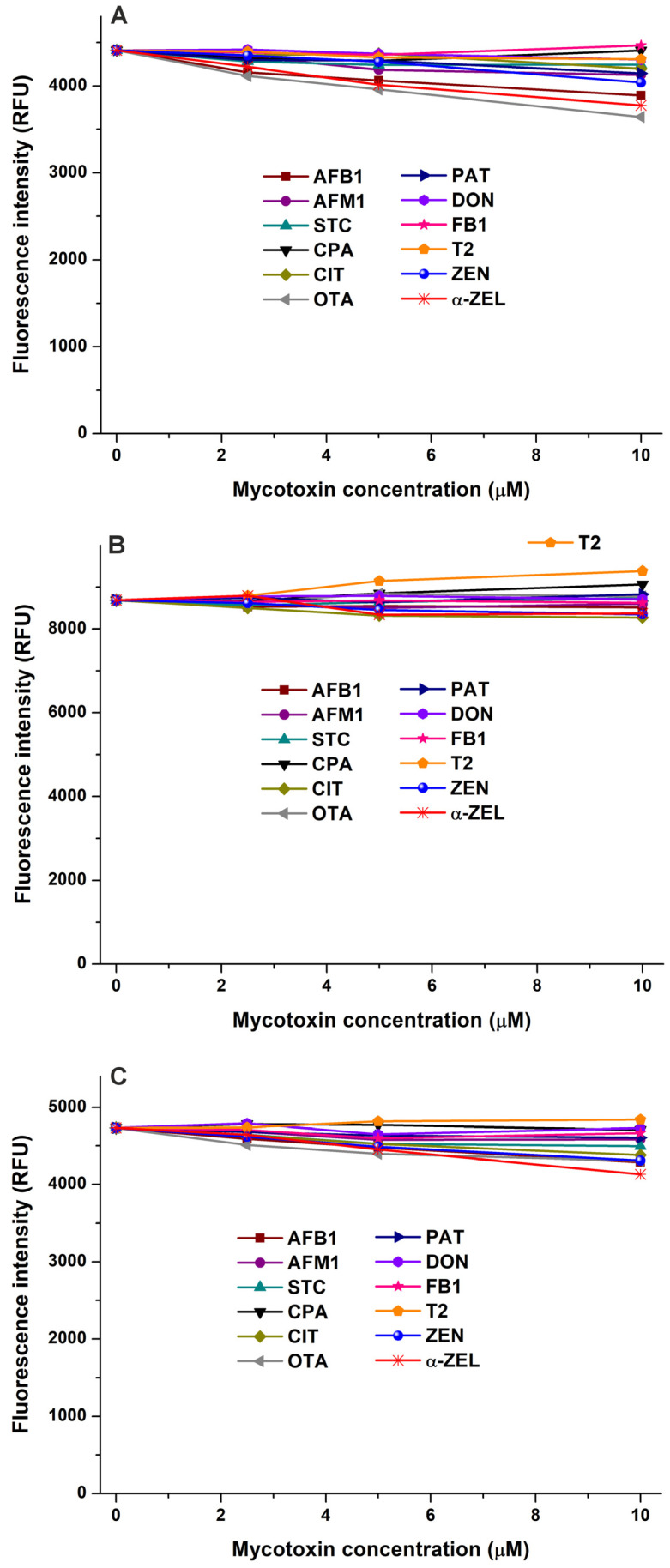
Effects of mycotoxins in fluorescence emission signals of casein ((**A**); CSN; λ_ex_ = 282 nm, λ_em_ = 342 nm), β-lactoglobulin ((**B**); LG; λ_ex_ = 282 nm, λ_em_ = 334 nm), and α-lactalbumin ((**C**); LA; λ_ex_ = 282 nm, λ_em_ = 332 nm) in sodium phosphate buffer (0.05 M, pH 6.8; protein concentration: each 2 μM; mycotoxin concentrations: 0, 2.5, 5, and 10 μM). Inner-filter effects of mycotoxins have been corrected (AFB1, aflatoxin B1; AFM1, aflatoxin M1; STC, sterigmatocystin; CPA, cyclopiazonic acid; CIT, citrinin; OTA, ochratoxin A; PAT, patulin; DON, deoxynivalenol; FB1, fumonisin B1; T2, T-2 toxin; ZEN, zearalenone; and α-ZEL, α-zearalenol).

To test the potential interactions of mycotoxins with CSN, LG, and LA, we examined their fluorescence quenching effects on these proteins. Due to their known appearance in bovine milk [3,4,5], two mycotoxin metabolites, AFM1 and α-ZEL, were also involved in these studies. The representative emission spectra of CSN, LG, and LA in the presence of mycotoxins are demonstrated in the Supplementary Materials (in Figure S5, Figure S6, and Figure S7, respectively). Most of the mycotoxins examined caused negligible changes in the emission signal of CSN, and slight decreases were noticed in the presence of AFB1, α-ZEL, and OTA (Figure 7A). Mycotoxins barely modified the emission intensity of LG; only T2 induced a minor increase (Figure 7B). Furthermore, we noticed no or only slight (AFB1, OTA, ZEN, and α-ZEL) quenching effects of mycotoxins on the emission signal of LA. These observations suggest that the mycotoxins examined likely do not form or form only low-stability complexes with these proteins. However, fluorescence quenching is an indirect technique; therefore, to confirm these results and to achieve a deeper insight, we also performed ultracentrifugation experiments.

In ultracentrifugation experiments, we selected STC, AFB1, AFM1, ZEN, and α-ZEL to examine their potential interactions with CSN, LG, and LA. In addition, BSA is the third most abundant whey protein in bovine milk [15], and the complex formation of serum albumins with STC, AFB1, AFM1, ZEN, and α-ZEL have been demonstrated in earlier studies [5,9,21]; therefore, we decided to also involve BSA in these experiments.

**Figure 8:**
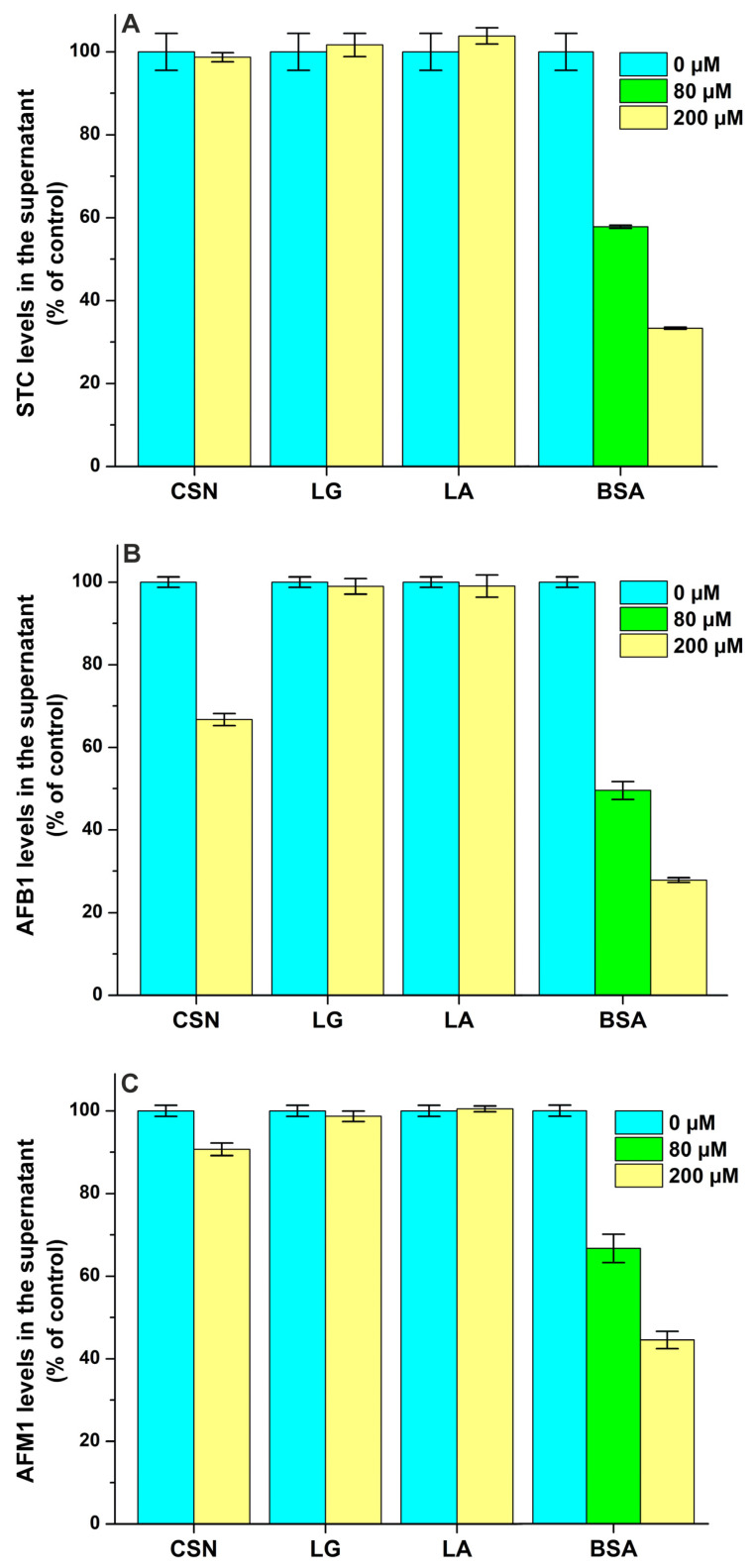

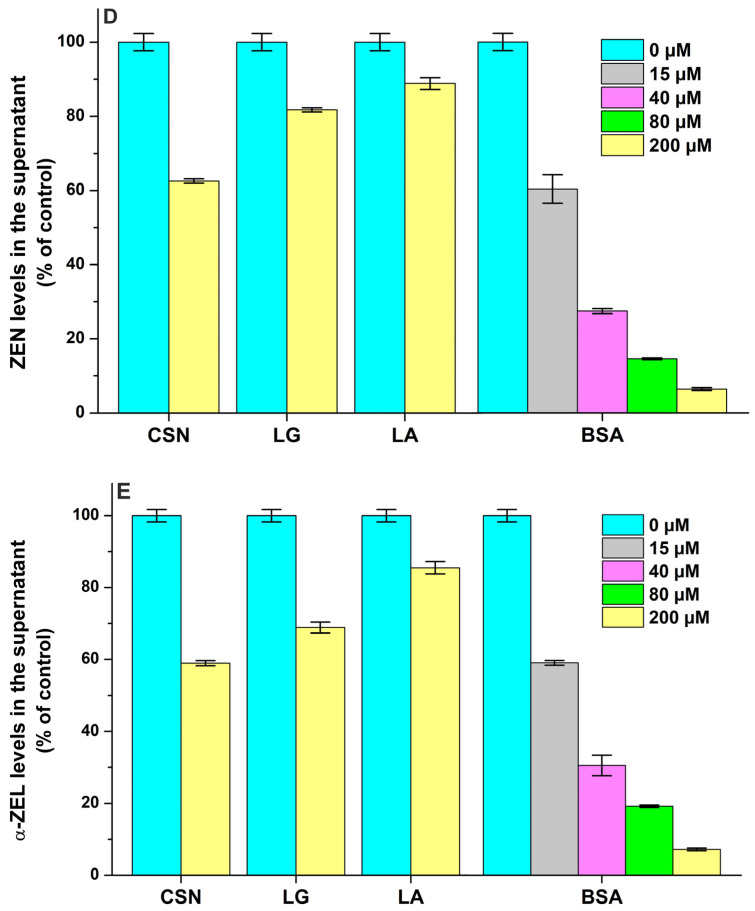
Effects of casein (CSN), β-lactoglobulin (LG), α-lactalbumin (LA), and bovine serum albumin (BSA) on the free concentrations of sterigmatocystin ((**A**); STC), aflatoxin B1 ((**B**); AFB1), aflatoxin M1 ((**C**); AFM1), zearalenone ((**D**); ZEN), and α-zearalenol ((**E**); α-ZEL) based on ultracentrifugation studies. Mycotoxin (each 2 μM) levels in the supernatant after ultracentrifugation in the absence and presence of milk proteins in sodium phosphate buffer (0.05 M, pH 6.8).

STC levels in the supernatant were not influenced by CSN, LG, and LA even at a 100-fold concentration compared to the ligand (Figure 8A), suggesting that STC does not form complexes with these proteins. However, we found a moderate interaction of STC with BSA (log*K* = 4.0), which was a little bit lower compared to the previously reported binding constant of the STC–HSA complex (log*K* = 4.3) [21].

We found the weak interaction of AFB1 with CSN (log*K* = 3.3), while LG and LA did not modify the free fraction of the mycotoxin (Figure 8B). In addition, ultracentrifugation studies demonstrated the moderate interaction of AFB1 with BSA (log*K* = 4.1), suggesting a somewhat lower binding constant than the one determined previously in fluorescence quenching studies (log*K* ≈ 4.5) [27,28]. Compared to AFB1, AFM1 formed less stable complexes with both CSN (log*K* = 2.7) and BSA (log*K* = 3.8), and it also did not show interactions with LG and LA (Figure 8C). Even if AFM1 binds to BSA with a 10-fold larger affinity than to CSN, the latter protein appears at more than a 100-fold higher molar concentration in the bovine milk. Therefore, it is unlikely that the stronger interaction of AFM1 with whey proteins can cause the accumulation of this mycotoxin in the whey fraction.

Each protein tested induced decreases in the free concentrations of ZEN (Figure 8D) and α-ZEL (Figure 8E). Based on these data, ZEN and α-ZEL form weak complexes (log*K* = 2.8 to 3.5) with CSN, LG, and LA (Table 2). However, the moderate interactions of ZEN (log*K* = 4.8) and α-ZEL (log*K* = 4.7) were noticed with BSA; which is in agreement with the previously reported binding constants of ZEN–BSA (log*K* = 4.8) and α-ZEL–BSA (log*K* = 4.5) complexes based on fluorescence quenching studies [9]. Thus, CSN forms more stable complexes with ZEN and α-ZEL compared to LG and LA, while the binding constants of BSA complexes are approximately 20-fold higher vs. the CSN complexes (Table 2). Nevertheless, we have to emphasize, again, the much higher molar concentrations of CSN (exceeding 1 mM) in bovine milk compared to LG (≈200 μM), LA (≈80 μM) and BSA (≈7 μM).

### 2.3. Limitations

In this section, we aimed to shortly summarize the most important limitations of our study. (1) The in vitro experiments applied provide a good starting point to the better understanding of the toxicokinetics and protein interactions of mycotoxins; however, further investigations (including in vivo evaluation) are reasonable to confirm the potential importance of these mycotoxin–protein interactions. (2) Fluorescence quenching is an indirect technique, which was applied in the current study to help the identification of the most relevant mycotoxin–protein interactions. The formation of ligand–protein complexes typically influences the fluorescence of tryptophane and/or tyrosine amino acids, resulting most commonly in decreases in the emission signal of the protein. From this point of view, the distance of the ligand binding site and the fluorescent amino acids seems to be highly important, and the emission signals of more than one tryptophan/tyrosine can be affected by ligand binding. As another weakness of quenching studies, higher concentrations of the ligand are applied vs. the protein, which may cause its interaction with low-affinity and/or nonspecific binding sites. Therefore, the binding constants determined based on fluorescence quenching experiments can be less accurate (even if this technique can commonly provide a good approximation). In addition, quenching studies alone do not provide solid evidence regarding the absence or presence of complex formations; thus, confirmatory measurements with other models/techniques should be performed. We also examined mycotoxin–protein interactions based on protein-induced fluorescence enhancement and/or ultracentrifugation experiments. Measurement of the AGP-induced increases in the emission signal of the ligands helped us to further confirm ZEN–AGP and ZEL–AGP interactions and to estimate the binding constants of these complexes. Even if it is also an indirect approach, we consider it as a more reliable technique compared to fluorescence quenching, because we can focus on the changes in the emission signal of one fluorophore (the ligand itself). Nevertheless, to use this strategy, the ligand is needed to exert relevant intrinsic fluorescence. We see ultracentrifugation as the most reliable technique applied in the current study, because it makes possible to directly quantify the free fraction of the ligand after the gentle sedimentation of the protein with the bound ligand molecules. Obviously, this latter technique also has disadvantages: it is very time-consuming, only a limited number of samples can be centrifuged at the same time, and the long centrifugation process cannot be successfully applied if the ligand has a low physicochemical stability. (3) We used our experimental models to explore some possibly important mycotoxin–protein interactions; nevertheless, the binding constants of these complexes can be affected by the changes in microenvironmental conditions (e.g., temperature and/or ionic strength).

## 3. Conclusions

In the current study, the interactions of mycotoxins (and some of their metabolites) with human AGP and bovine milk proteins (CSN, LG, LA, and BSA) were examined. Both fluorescence spectroscopic and ultracentrifugation experiments demonstrated the formation of highly stable complexes of AGP with ZEN, ZELs, and STC, recommending its potentially important role in the plasma protein binding of these mycotoxins. We observed no or only weak interactions of mycotoxins with CSN, LG, and LA. Furthermore, AFM1, ZEN, and α-ZEL bound with a higher affinity to CSN compared to the two major whey proteins, LG and LA. The binding constants of AFM1–BSA, ZEN–BSA, and α-ZEL–BSA were considerably higher compared to the corresponding mycotoxin–CSN complexes. However, due to the relatively low levels of BSA in bovine milk, it likely has minor importance. Also considering the limitations of our in vitro explorative study, further investigations are desirable to evaluate the relevance of these interactions.

## 4. Materials and Methods

### 4.1. Reagents

Aflatoxin B1 (AFB1), sterigmatocystin (STC), cyclopiazonic acid (CPA), citrinin (CIT), ochratoxin A (OTA), patulin (PAT), deoxynivalenol (DON), fumonisin B1 (FB1), T-2 toxin (T2), zearalenone (ZEN), α-zearalenol (α-ZEL), β-zearalenol (β-ZEL), α_1_-acid glycoprotein (AGP, human), α-lactalbumin (LA, bovine), β-lactoglobulin (LG, bovine), and bovine serum albumin (BSA) were obtained from Merck (Darmstadt, Germany). Aflatoxin M1 (AFM1; Apollo Scientific, Cheshire, UK) and casein (CSN, bovine; Thermo Fisher Scientific, Waltham, MA, USA) were used as received. HPLC-grade methanol and acetonitrile were purchased from Molar Chemicals (Halásztelek, Hungary). Other chemicals were analytical or spectroscopic grade. Stock solutions of mycotoxins (each 5 mM) were prepared in dimethyl sulfoxide and stored at −20 °C.

### 4.2. Fluorescence Spectroscopic Studies

Fluorescence studies were carried out with a Hitachi F-4500 (Hitachi Ltd., Tokyo, Japan) fluorometer, applying standard 1 cm × 1 cm cuvettes and 90-degree fluorescence detection. We did not use direct temperature control in the instrument; however, the temperature in the laboratory was documented in the 22–24 °C range. Before the fluorescence spectra were recorded, at least 30 min equilibration time of the samples was allowed. Confirmatory measurements were performed after 60 min and 180 min, where we did not see differences compared to the data observed after 30 min, demonstrating that the equilibrium was established. Fluorescence spectroscopic experiments were performed in triplicates. The following excitation/emission maxima were determined and applied for the evaluation of the protein signals: 285/337 nm for AGP, 282/342 nm for CSN, 282/334 nm for LG, and 282/332 nm for LA. In quenching studies, the inner-filter effects of mycotoxins were corrected based on the absorbance of mycotoxins (see representative absorption spectra in Figure S8) determined with a Hitachi U-3900 UV-Vis spectrophotometer (Hitachi Ltd., Tokyo, Japan), applying the following equation [29]:

(1) $$F_{cor}=F_{obs}\times 10^{(A_{ex}+A_{em})/2}$$

where *F_cor_* and *F_obs_* are the corrected and the measured fluorescence emission intensities of the protein, respectively, while *A_ex_* and *A_em_* are the absorbance values of mycotoxins at the excitation and emission wavelength used, respectively.

The fluorescence emission spectra of AGP (1 μM) were collected in the presence of increasing mycotoxin concentrations (0–5 μM). To approximate the extracellular physiological microenvironment, mycotoxin–AGP interactions were studied in phosphate-buffered saline (PBS, pH 7.4). Thereafter, we also examined the changes in the emission signals of ZEN and ZELs (each 1 μM) in the presence of increasing AGP levels (0–5 μM), where the excitation wavelength of these mycotoxins was applied (λ_ex_ = 315 nm) [24]. Based on these data, the binding constants (*K*; unit: L/mol) of mycotoxin–protein complexes were determined with non-linear fitting applying the Hyperquad2006 program, as it has been previously reported [9,27]. During this evaluation, we tested 1:1 and 1:2 stoichiometries of the complexes, but we found assessable results only with the 1:1 model.

The mycotoxin-induced (0–10 μM) changes in the emission signals of CSN, LG, and LA (each 2 μM) were examined in sodium phosphate buffer (0.05 M, pH 6.8), because the pH of fresh bovine milk is typically in the 6.5–6.9 range [30]. In quenching studies, we applied no more than fivefold ligand concentrations vs. the proteins examined because of the following two reasons: (1) It typically induces relevant changes in the emission signal of the protein if the formed complex is relatively stable (≥10^4^ L/mol) [9,10,27] and (2) the presence of excessively high levels of ligand molecules (compared to the protein) may lead to their interactions with low-affinity/nonspecific binding sites (while, in the reality, the molar concentrations of AGP in the human circulation and major milk proteins in bovine milk highly exceed mycotoxin levels).

### 4.3. Ultracentrifugation Studies

Similarly to the spectroscopic studies, the interactions of mycotoxins with AGP were evaluated in PBS (pH 7.4), while sodium phosphate buffer (0.05 M, pH 6.8) was applied regarding CSN, LG, LA, and BSA. The proteins (with the bound ligand molecules) were sedimented by ultracentrifugation [21,22]. Mycotoxins (each 1 μM) without or with the proteins examined (0–200 μM) were centrifuged for 16 h (170,000× *g*, 20 °C) applying an Optima MAX-XP ultracentrifuge (Beckman Coulter, Brea, CA, USA), then the free fraction of the ligand was directly quantified with LC-MS or HPLC-UV (see details in Section 4.4 and Section 4.5) in the protein-free supernatants. Binding constants (*K*) of mycotoxin–protein complexes were calculated assuming 1:1 stoichiometry [21]:

(2) $$K=\frac{\left[MP\right]}{\left[M\right]\times [P]}$$

where [*M*] is the molar concentration of the free (not protein-bound) mycotoxin, [*P*] is the molar concentration of the unbound protein, and [*MP*] is the molar concentration of the mycotoxin–protein complex. After ultracentrifugation, the recovery was above 90% regarding each mycotoxin tested.

### 4.4. LC–MS Analyses of ZEN, α-ZEL, and β-ZEL

After ultracentrifugation of ZEN, α-ZEL, and β-ZEL with AGP, supernatants were diluted with equal volume of acetonitrile then injected into the LC-MS without any further preparation steps. ZEN and ZEL concentrations were determined using a Prominence HPLC system coupled with a LCMS-2020 single quadrupole MS (Shimadzu, Kyoto, Japan). The analytes were separated using reverse phase gradient elution on a Kinetex XB-C18 column (100 mm × 2.1 mm, 2.6 µm particle size; Phenomenex, Torrance, CA, USA), with a flow rate of 0.3 mL/min and column temperature of 40 °C. The injected sample volume was 10 µL. Eluent A was 0.1 *v*/*v*% formic acid and 5 mM ammonium formate in water, while eluent B was 0.1 *v*/*v*% formic acid in acetonitrile. The gradient profile was as follows: eluent B ratio, starting from 10% of B, was linearly increased to 100% of B within 8 min, followed by 3 min of washing by 100% of B, then re-equilibrating the column, decreasing to 10% of B, and holding it for 3 min (total run-time: 15 min).

For MS quantitative analysis, ESI ion source was used in negative ion mode. For each toxin analyzed, three different m/z values (1 for quantitative analysis and additional 2 for confirmation) were used, which were determined by injecting 100 mg/L mycotoxin standards in scan mode. These were as follows: 317 (quantitative), 318 and 635 (confirmatory) for ZEN; and 319 (quantitative), 320 and 639 (confirmatory) for α-ZEL and β-ZEL.

### 4.5. HPLC–UV Analyses

After the ultracentrifugation of AFB1, AFM1, STC, ZEN, and α-ZEL with milk proteins (CSN, LG, LA, and BSA) as well as STC with AGP, these mycotoxins were analyzed from the supernatants, using a integrated HPLC system (Jasco, Tokyo, Japan): autosampler (AS-4050), binary pump (PU-4180), and UV detector (UV-975). Chromatograms were evaluated with the ChromNAV2 software (Jasco, Tokyo, Japan). The supernatants of AFB1 and AFM1 were directly injected, while STC, ZEN, and α-ZEL samples were diluted with equal volume of acetonitrile before the HPLC–UV analyses.

AFB1, AFM1, and STC were quantified by applying the following HPLC methods. A 20 μL volume of samples was driven through a precolumn (Security Guard C18, 4.0 × 3.0 mm; Phenomenex, Torrance, CA, USA) linked to a Gemini C18 (150 × 4.6 mm, 5 μm; Phenomenex) analytical column. The isocratic elution was performed at room temperature, with 1 mL/min flow rate. The mobile phases contained water, methanol, and acetonitrile (45:35:25 *v*/*v*%) for AFB1; water, methanol, and acetonitrile (40:30:30 *v*/*v*%) for AFM1; and water and acetonitrile (40:60 *v*/*v*%) for STC. AFB1 and AFM1 were detected at 362 nm, and STC at 331 nm. The major validation parameters for AFB1 were the following: linearity (0.1–5.0 μM), *R*^2^ = 0.999; limit of detection (LOD, signal-to-noise ratio of 3), 0.1 μM; limit of quantification (LOQ, signal-to-noise ratio of 10), 0.3 μM; and intraday precision (*n* = 6), 3.4%. The major validation parameters for AFM1 were the following: linearity (0.1–5.0 μM), *R*^2^ = 0.999; LOD, 0.03 μM; LOQ, 0.1 μM; and intraday precision (*n* = 6), 0.9%. The major validation parameters for STC were the following: linearity (0.1–5.0 μM), *R*^2^ = 0.999; LOD, 0.02 μM; LOQ, 0.06 μM; and intraday precision (*n* = 6), 2.2%.

ZEN and α-ZEL were quantified applying the following HPLC method. A 20 μL volume of samples was driven through a precolumn (Security Guard C18, 4.0 × 3.0 mm; Phenomenex) linked to a Kinetex EVO-C18 (250 × 4.6 mm, 5 μm; Phenomenex) analytical column. The isocratic elution was performed at room temperature, with 1 mL/min flow rate, applying water, acetonitrile, and methanol (40:50:10 *v*/*v*%) in the mobile phase. ZEN and α-ZEL were detected at 274 nm. The major validation parameters for ZEN were the following: linearity (0.1–5.0 μM), *R*^2^ = 0.999; LOD, 0.02 μM; LOQ, 0.07 μM; and intraday precision (*n* = 6), 1.5%. The major validation parameters for α-ZEL were the following: linearity (0.1–5.0 μM), *R*^2^ = 0.999; LOD, 0.02 μM; LOQ, 0.05 μM; and intraday precision (*n* = 6), 1.2%.

### 4.6. Molecular Modeling Studies

ZEN, α-ZEL, β-ZEL, and STC were built in Maestro (Schrödinger Release 2024-1: Maestro, Schrödinger; New York, NY, USA) and a quick, local steepest descent energy minimization step was performed.

Atomic coordinates of the human AGP in complex with UCN-01 were obtained from the Protein Data Bank (PDB [31]) with PDB code 7oub [32]. This structure was selected for the present study, as UCN-01 has a similar multi-ring structure compared to the investigated mycotoxins of the present study. Hydrogens were added on the target protein; the bound ligand was removed prior to docking.

A grid box was centered on the average coordinates of the bound ligand (UCN-01 in PDB:7oub) at coordinates −6.07, 30.66, and −1.06 in AutoDock Tools [33]. The size of the box was set to 50 × 50 × 50 Å. Active torsions on the ligands were allowed, the target was treated as rigid structure. Ten docking runs were performed using Lamarckian genetic algorithm as in our earlier report [34]. The resultant binding modes were clustered into ranks based on their calculated free energy of binding (Δ*G_b_*); the lower rank corresponding to a better Δ*G_b_*. Regarding UCN-01, root mean squared deviation (RMSD) was calculated between the docked and the experimental binding mode. For the other ligands, this was not applicable.

## Figures and Tables

**Table 1:** Decimal logarithmic values of binding constants (log*K* ± SEM) of mycotoxin–AGP complexes based on fluorescence spectroscopic (*n* = 3) and ultracentrifugation (*n* = 5 to 6) studies (unit of *K* is L/mol), where means ± SEM were calculated from log*K* values determined in parallel experiments. Log*K* and SEM for individual non-linear fittings are represented in Table S1 regarding each parallel spectroscopic measurement.

Mycotoxin–Protein Complex	log*K* FL Quenching	log*K* FL Enhancement	log*K* Ultracentrifugation
STC–AGP	5.85 ± 0.01	–	5.55 ± 0.02
ZEN–AGP	5.90 ± 0.05	6.28 ± 0.06	6.43 ± 0.06
α-ZEL–AGP	5.65 ± 0.04	5.51 ± 0.05	5.48 ± 0.07
β-ZEL–AGP	5.78 ± 0.02	5.94 ± 0.06	5.73 ± 0.02

AGP, α_1_-acid glycoprotein; STC, sterigmatocystin; ZEN, zearalenone; α-ZEL, α-zearalenol; and β-ZEL, β-zearalenol.

**Table 2:** Decimal logarithmic values of the binding constants (log*K* ± SEM; *n* = 3 to 6) of mycotoxin–protein complexes based on ultracentrifugation studies (the unit of *K* is L/mol).

	CSN	LG	LA	BSA
**STC**	–	–	–	3.98 ± 0.01
**AFB1**	3.34 ± 0.03	–	–	4.11 ± 0.02
**AFM1**	2.70 ± 0.08	–	–	3.80 ± 0.03
**ZEN**	3.48 ± 0.01	3.05 ± 0.02	2.79 ± 0.08	4.79 ± 0.05
**α-ZEL**	3.54 ± 0.01	3.35 ± 0.03	2.92 ± 0.06	4.73 ± 0.02

AFB1, aflatoxin B1; AFM1, aflatoxin M1; STC, sterigmatocystin; ZEN, zearalenone; α-ZEL, α-zearalenol; CSN, casein; LG, β-lactoglobulin; LA, α-lactalbumin; and BSA, bovine serum albumin.

## Data Availability

The original contributions presented in this study are included in the article and Supplementary Materials. Further inquiries can be directed to the corresponding authors.

## Supplementary Materials

The following supporting information can be downloaded at https://www.mdpi.com/article/10.3390/toxins17040151/s1, Figure S1: Representative fluorescence emission spectra of AGP in the presence of mycotoxins; Figure S2: Evaluation of fluorescence emission data of quenching experiments with the Hyperquad2006 software; Figure S3: Evaluation of emission intensity data of fluorescence enhancement experiments with the Hyperquad2006 software; Table S1: Log*K* and SEM values for individual non-linear fittings; Figure S4: Structural match between the re-docked and experimental binding mode of UCN-01; Figure S5: Representative fluorescence emission spectra of CSN in the presence of mycotoxins; Figure S6: Representative fluorescence emission spectra of LG in the presence of mycotoxins; Figure S7: Representative fluorescence emission spectra of LA in the presence of mycotoxins; and Figure S8: Representative absorption spectra of the proteins and mycotoxins examined.

## Abbreviations

The following abbreviations are used in this manuscript:

AFB1	aflatoxin B1
AFM1	aflatoxin M1
AGP	α_1_-acid glycoprotein
BSA	bovine serum albumin
CAS	casein
CIT	citrinin
CPA	cyclopiazonic acid
DON	deoxynivalenol
FB1	fumonisin B1
HSA	human serum albumin
LA	α-lactalbumin
LG	β-lactoglobulin
OTA	ochratoxin A
PAT	patulin
RFU	relative fluorescence unit
STC	sterigmatocystin
T2	T-2 toxin
ZEN	zearalenone
α-ZEL	α-zearalenol
β-ZEL	β-zearalenol
