# Supplementary material for: Interaction of Mycotoxins with α1-Acid Glycoprotein (AGP) and Bovine Milk Proteins: Zearalenone, Zearalenols, and Sterigmatocystin Form Highly Stable Complexes with AGP
Source: Toxins (Basel). 2025 Mar 21;17(4):151. doi: 10.3390/toxins17040151 (PMC12031583; doi:10.3390/toxins17040151)
Supplement: Supplementary file 1 [file toxins-17-00151-s001.zip › toxins-3481351-supplementary.pdf]

Interaction of Mycotoxins with α_1-Acid Glycoprotein (AGP) and Bovine Milk Proteins: Zearalenone, Zearalenols, and Sterigmatocystin Form Highly Stable Complexes with AGP

SUPPLEMENTARY MATERIALS

Miklós Poór [1,2,*], Patrik Gömbös [3], András Szabó [3,4], Balázs Zoltán Zsidó [5], Csaba Hetényi [5,6], Tamás Huber [7], András Lukács [7] and Sándor Kunsági-Máté [8,9]

[1] Department of Laboratory Medicine, Medical School, University of Pécs, Ifjúság útja 13, H-7624 Pécs, Hungary
[2] Molecular Medicine Research Group, János Szentágothai Research Centre, University of Pécs, Ifjúság útja 20, H-7624 Pécs, Hungary
[3] Institute of Physiology and Nutrition, Department of Physiology and Animal Health, Agribiotechnology and Precision Breeding for Food Security National Laboratory, Hungarian University of Agriculture and Life Sciences, H-2103 Gödöllő, Hungary; gombos.patrik@uni-mate.hu (P.G.);
szabo.andras@uni-mate.hu (A.S.)
[4] HUN-REN-MATE Mycotoxins in the Food Chain Research Group, Hungarian University of Agriculture and Life Sciences, Guba Sándor u. 40, H-7400 Kaposvár, Hungary
[5] Pharmacoinformatics Unit, Department of Pharmacology and Pharmacotherapy, Medical School, University of Pécs, Szigeti út 12, H-7624 Pécs, Hungary; zsido.balazs@pte.hu (B.Z.Z.); hetenyi.csaba@pte.hu (C.H.)
[6] National Laboratory for Drug Research and Development, H-1117 Budapest, Hungary
[7] Department of Biophysics, Medical School, University of Pécs, Szigeti út 12, H-7624 Pécs, Hungary; tamas.huber@aok.pte.hu (T.H.); andras.lukacs@aok.pte.hu (A.L.)
[8] Department of Organic and Medicinal Chemistry, Faculty of Pharmacy, University of Pécs, Honvéd u. 1,
H-7624 Pécs, Hungary; kunsagi-mate.sandor@gytk.pte.hu
[9] Green Chemistry Research Group, János Szentágothai Research Centre, University of Pécs, Ifjúság útja 20, H-7624 Pécs, Hungary
* Correspondence: poor.miklos@pte.hu

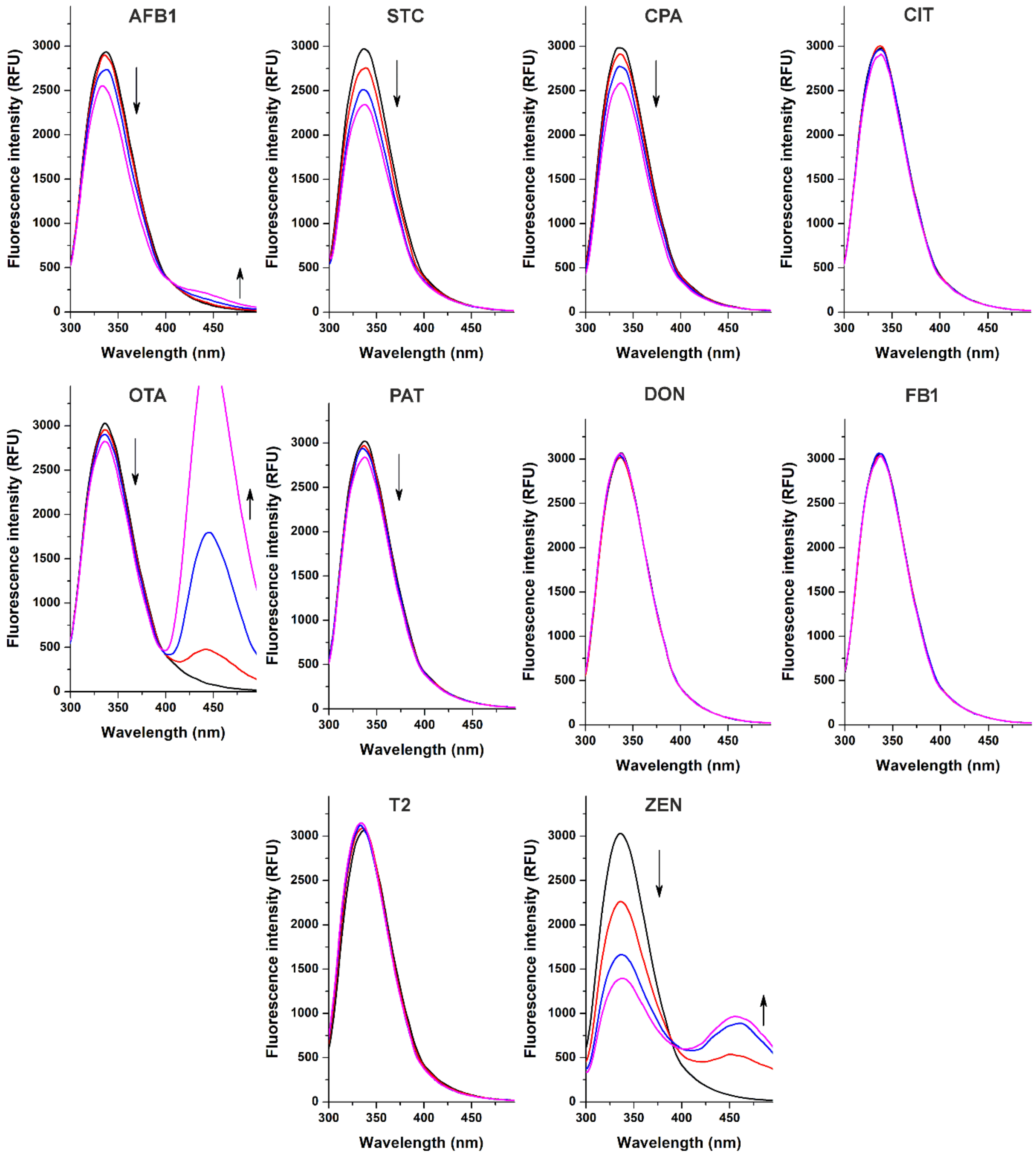

Figure S1. Representative fluorescence emission spectra of AGP (1 μM) in the presence mycotoxins (0, 0.5, 2, and 5 μM) in PBS (pH 7.4; λ_{ex} = 285 nm).

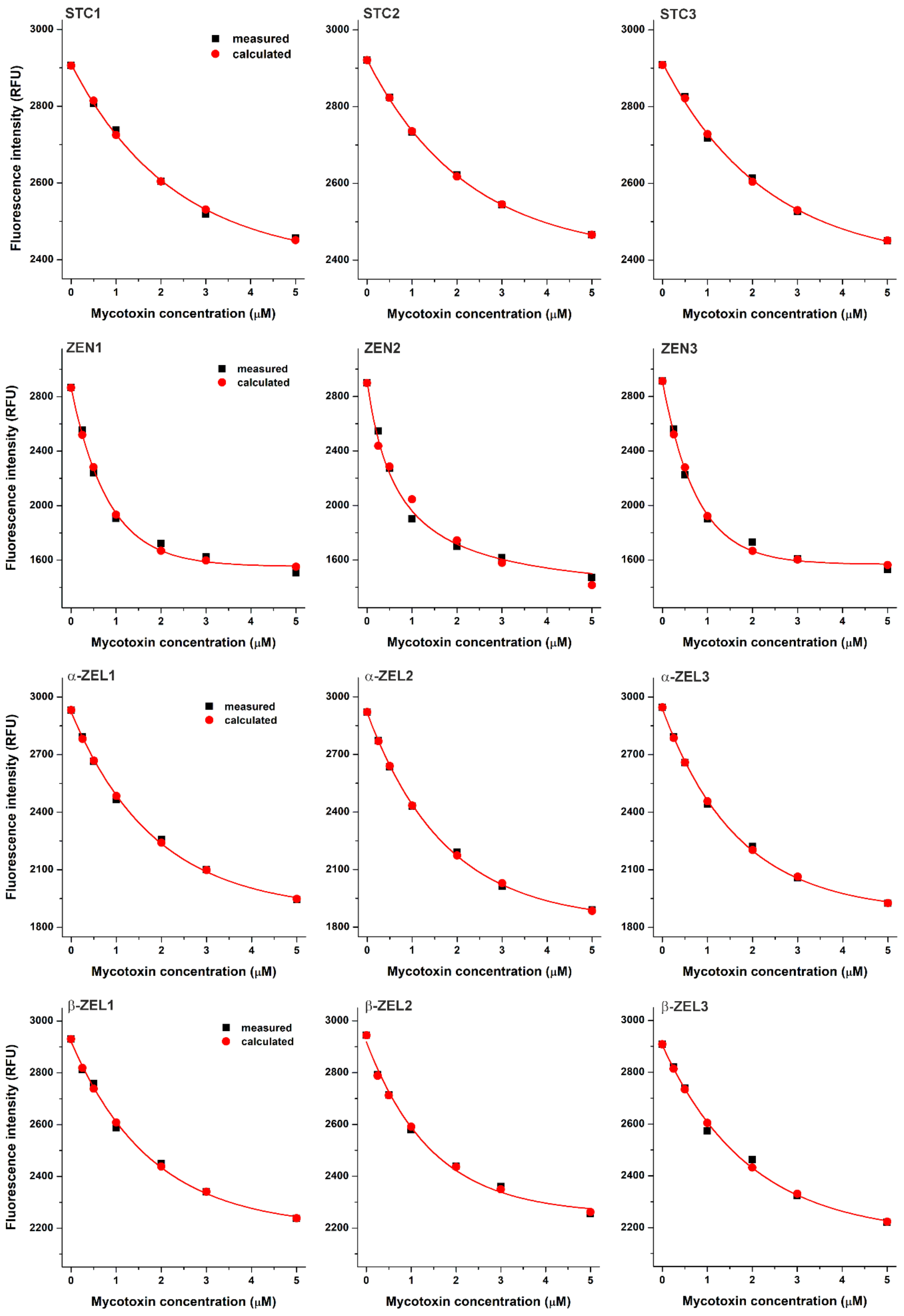

Figure S2. Evaluation of fluorescence quenching experiments (1 μM AGP + 0–5 μM mycotoxins in PBS; parallels 1, 2 and 3): measured emission intensity data (black) vs. data calculated by the Hyperquad software (red).

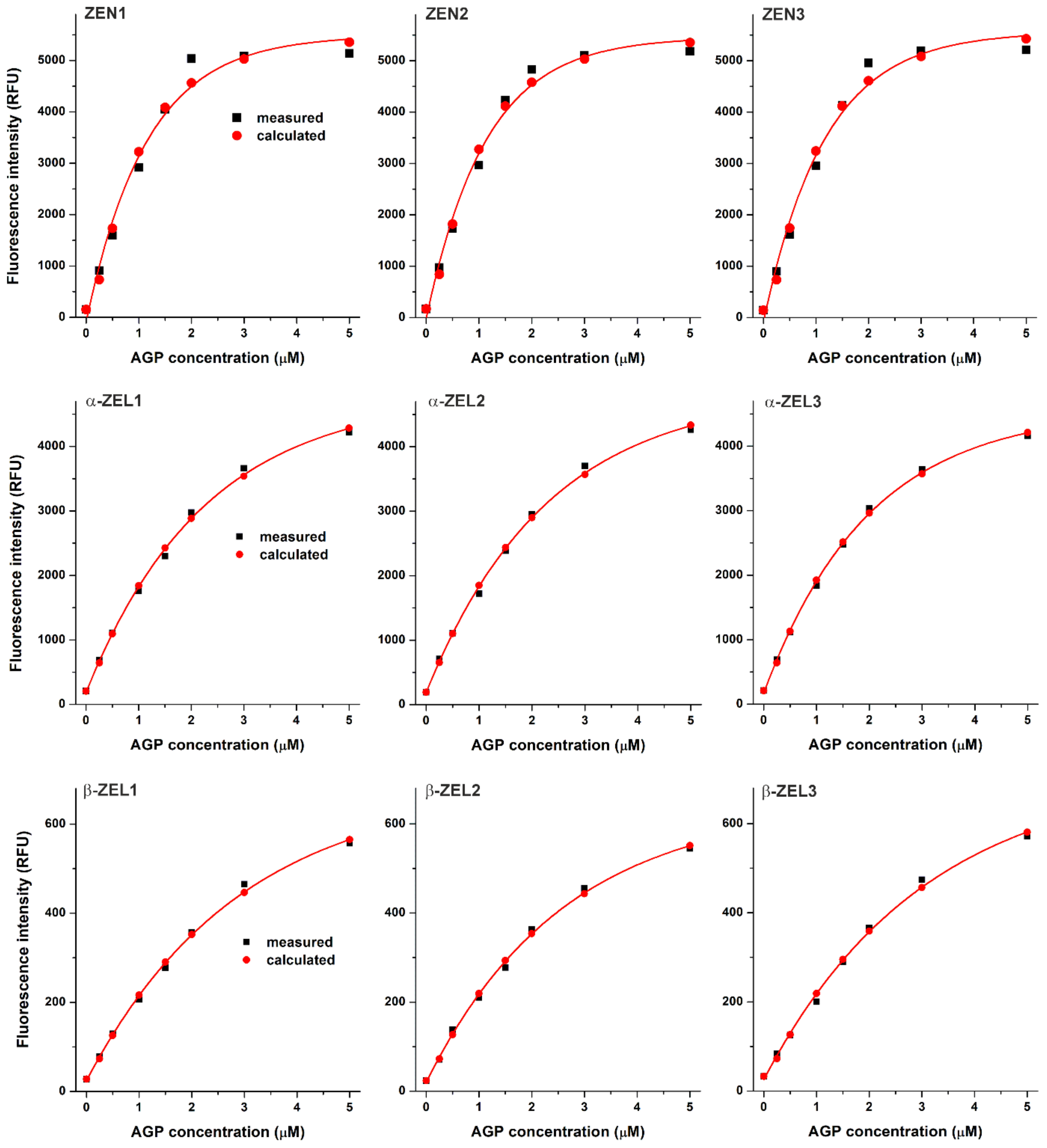

Figure S3. Evaluation of fluorescence enhancement experiments (1 μM ZEN/ZEL + 0–5 μM AGP in PBS; parallels 1, 2 and 3): measured emission intensity data (black) vs. data calculated by the Hyperquad software (red).

Table S1. The log*K* and SEM values for the individual non-linear fittings with the Hyperquad software regarding each parallel spectroscopic measurement.

Mycotoxin–protein complex	log*K* ± SEM 1.	log*K* ± SEM 2.	log*K* ± SEM 3.
STC–AGP (FL quenching)	5.84 ± 0.11	5.82 ± 0.03	5.87 ± 0.08
ZEN–AGP (FL quenching)	5.87 ± 0.06	5.84 ± 0.17	6.00 ± 0.13
α-ZEL–AGP (FL quenching)	5.57 ± 0.07	5.67 ± 0.05	5.70 ± 0.06
β-ZEL–AGP (FL quenching)	5.76 ± 0.06	5.82 ± 0.09	5.76 ± 0.08
ZEN–AGP (FL enhancement)	6.18 ± 0.04	6.39 ± 0.08	6.26 ± 0.08
α-ZEL–AGP (FL enhancement)	5.46 ± 0.06	5.46 ± 0.08	5.62 ± 0.07
β-ZEL–AGP (FL enhancement)	5.83 ± 0.04	5.94 ± 0.16	6.04 ± 0.11

AGP, α_1-acid glycoprotein; STC, sterigmatocystin; ZEN, zearalenone; α-ZEL, α-zearalenol; β-ZEL, β-zearalenol

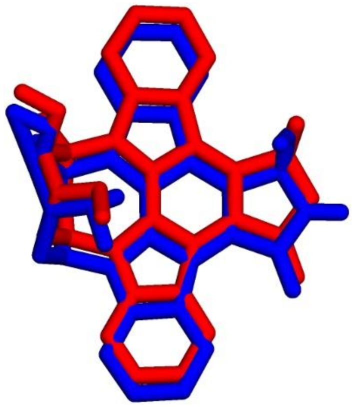

Figure S4. Structural match between the re-docked (blue sticks) and experimental (red sticks) binding mode of UCN-01.

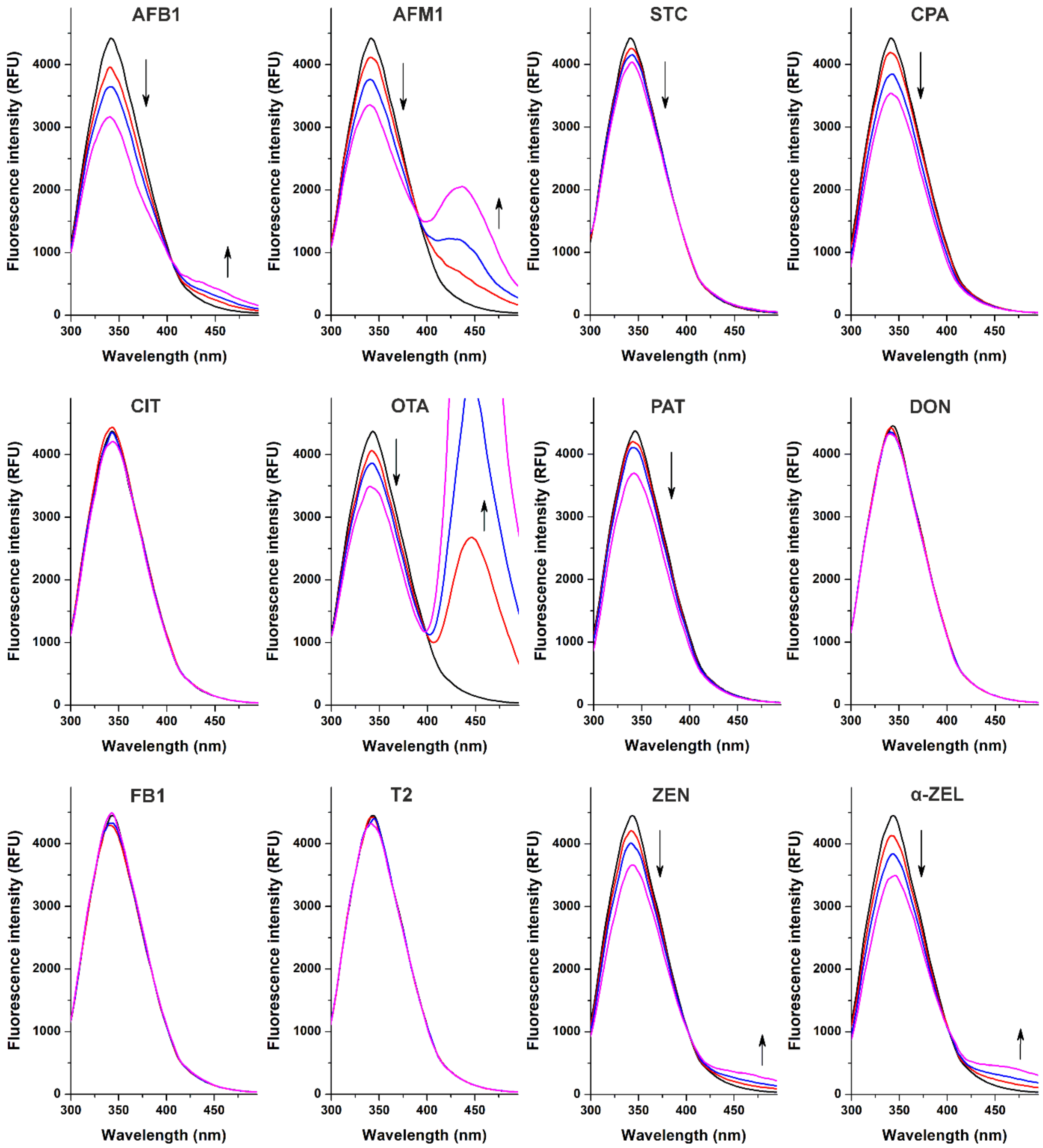

Figure S5. Representative fluorescence emission spectra of CSN (2 μM) in the presence mycotoxins (0, 2.5, 5, and 10 μM) in PBS (pH 7.4; λ_{ex} = 282 nm).

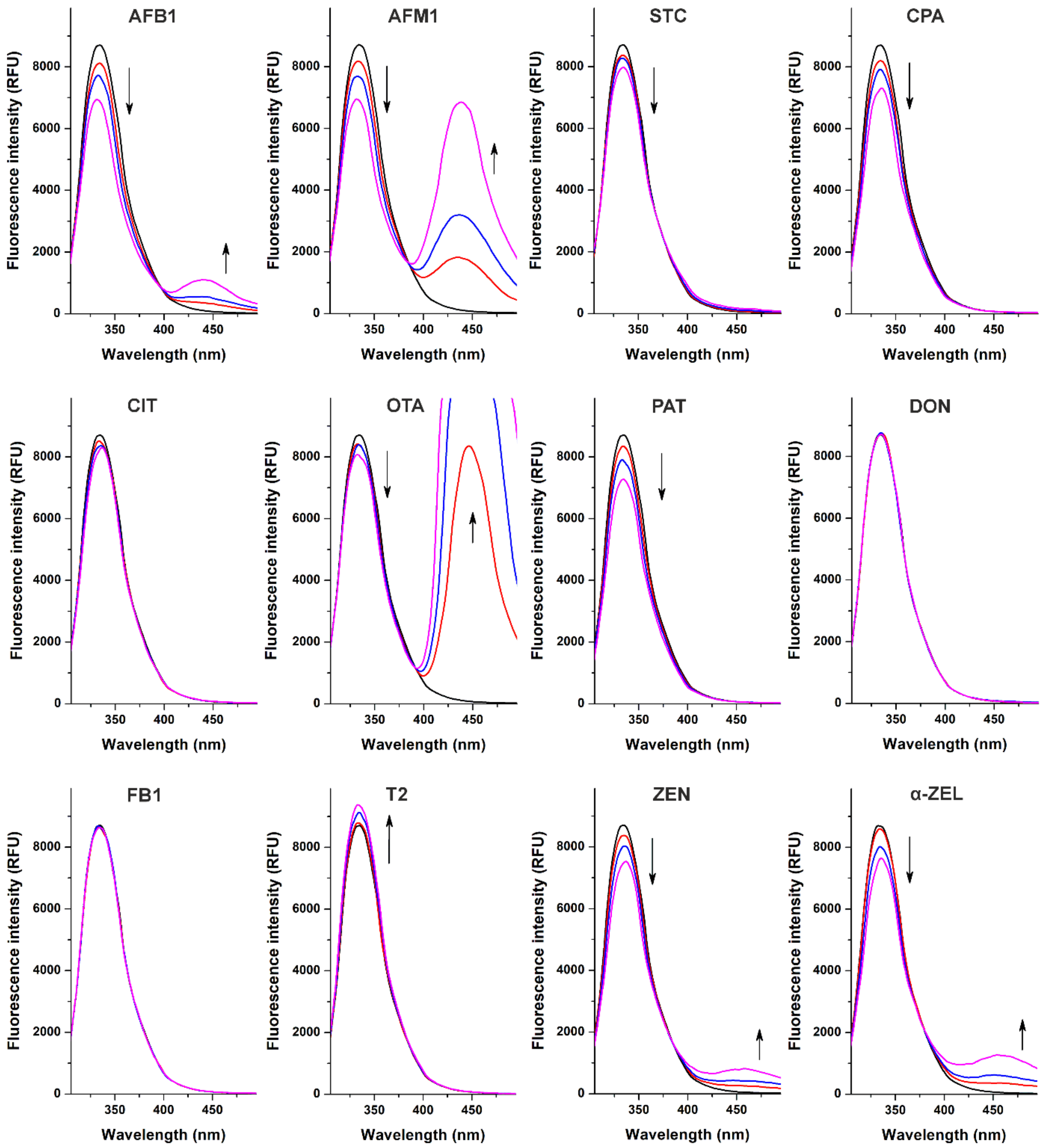

Figure S6. Representative fluorescence emission spectra of LG (2 μM) in the presence mycotoxins (0, 2.5, 5, and 10 μM) in PBS (pH 7.4; λ_{ex} = 282 nm).

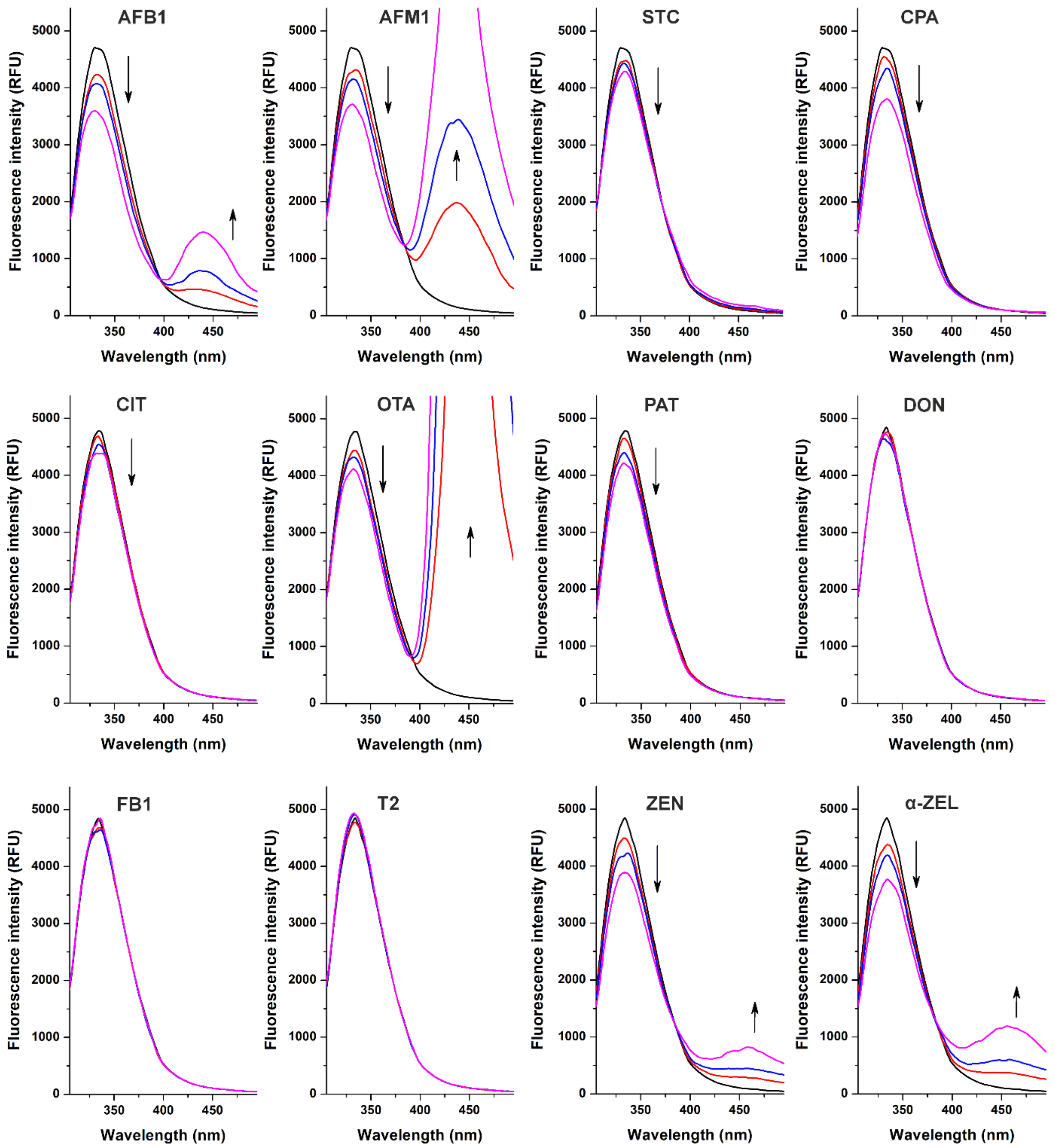

Figure S7. Representative fluorescence emission spectra of LA (2 μM) in the presence mycotoxins (0, 2.5, 5, and 10 μM) in PBS (pH 7.4; λ_{ex} = 282 nm).

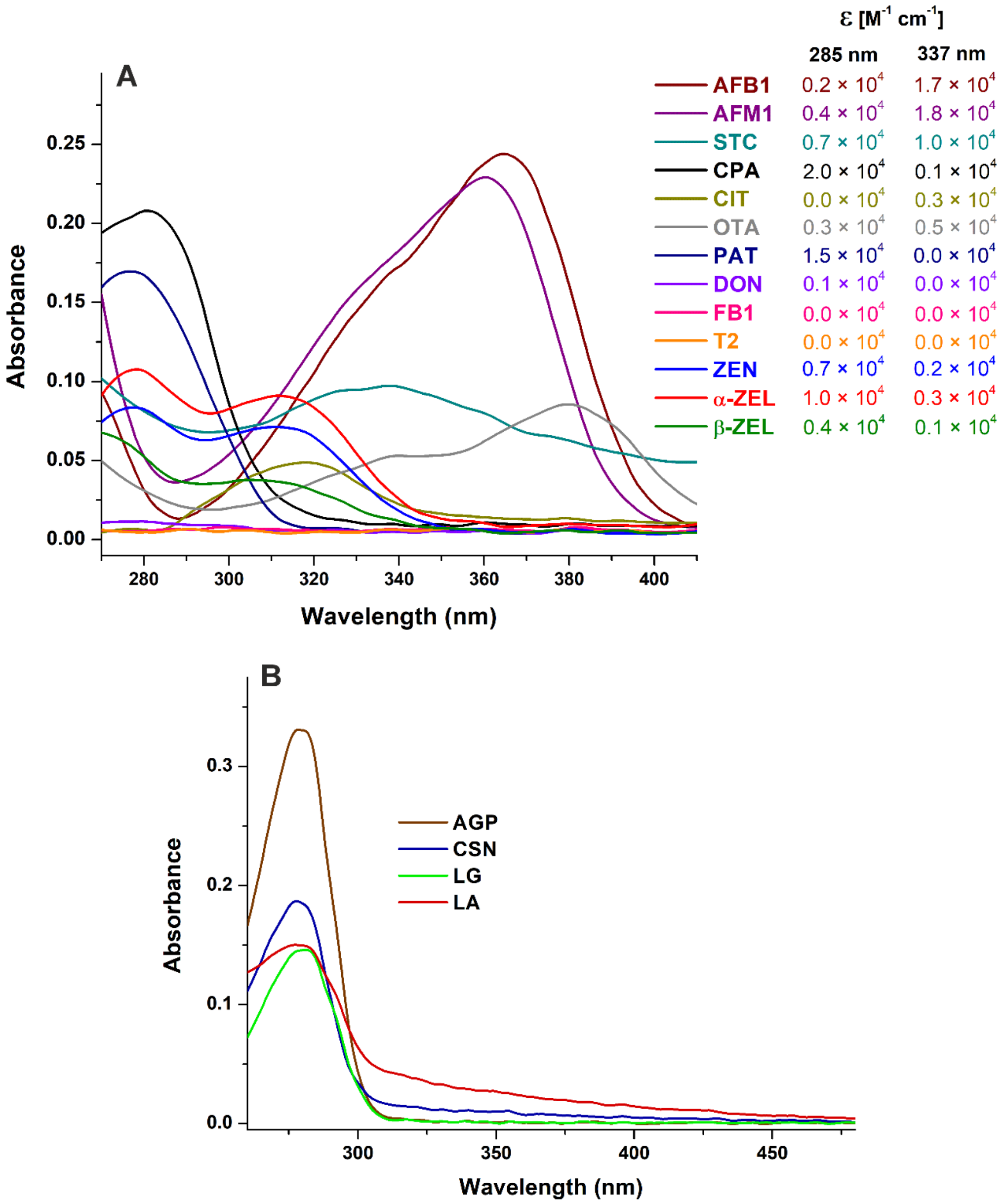

Figure S8. (**A**) Absoption spectra of mycotoxins (each 10 μM) in PBS (pH 7.4), and their molar extinction coefficients (ε) at 285 and 337 nm. (**B**) Absoption spectra of AGP (10 μM) in PBS (pH 7.4) and milk proteins (CSN, LG, and LA; each 10 μM) in sodium phosphate buffer (0.05 M, pH 6.8).
